# Impact of temperament on mental illness stigma among medical students

**DOI:** 10.1192/j.eurpsy.2022.553

**Published:** 2022-09-01

**Authors:** L. Brahmi, B. Amemou, A. Adouni, A. Mhalla, L. Gaha

**Affiliations:** Fattouma Bourguiba University Hospital, Psychiatry Department, Monastir, Tunisia

**Keywords:** stigma, medical student, temperament

## Abstract

**Introduction:**

Mental illness stigma is the most significant obstacle impeding the wellbeing of individuals with such conditions. Thus, research on determinants of mental illness stigma may be of crucial importance in avoiding these attitudes. Affective temperaments are thought to be present in up to 20% of the healthy general population. However, there are very few studies addressing the relationship between temperament and mental health-related stigma.

**Objectives:**

Evaluate attitudes and behavioral responses of medical students towards individuals with a mental illness. Explore factors associated with stigma including temperament.

**Methods:**

A cross-sectional study was conducted among students in medical universities.

All participants were invited to complete a brief anonymous electronic survey administered on the google forms online platform.

Data were collected using self-administered questionnaires, Stigma Measurement, Mental Illness: Clinicians’ Attitudes (MICA). Students were also asked to complete the TEMPS-A Scale.

**Results:**

The sample consisted of 1028 respondents (9.3% of the total population). Females represented 78,3% of the study sample. A dominant affective temperament was found in 17% of the cases under study, represented mainly by depressive and irritable temperaments. Bivariate correlations performed to assess the association between temperament and mental illness stigma revealed that a positive relationship was identified between the MICA scale and hyperthymic temperament( p=0,04). There were no significant associations between the other type of temperaments and The MICA scale.

**Conclusions:**

Students’ temperament should be considered in developing anti-stigma programs in undergraduate education. Further researches should be undertaken to disentangle the complex relationship among demographic features, personality traits, and attitudes toward people with a mental illness.

**Disclosure:**

No significant relationships.

